# Pediatric Electrophysiology in 2024: End of the Year Review

**DOI:** 10.19102/icrm.2025.16014

**Published:** 2025-01-15

**Authors:** Johannes C. Von Alvensleben, Kathryn K. Collins

**Affiliations:** 1 Section of Electrophysiology, Division of Cardiology, Department of Pediatrics, Children’s Hospital Colorado, University of Colorado School of Medicine, Aurora, CO, USA

**Keywords:** Cardiac ablation, biological pacemaker, congential heart disease, leadless pacing, supraventricular tachycardia

In 2024, research in pediatric patients and those with congenital heart disease (CHD) focused on advancements in device technology, safety considerations, long-term outcomes, and specific challenges related to these specialized populations. Below is a summary of key findings from recent studies.

## Cardiac ablation: personalized approaches, effectiveness, and safety concerns

The push to avoid fluoroscopy during ablation has been buoyed by advances in electroanatomic mapping (EAM) systems. While admittedly accurate, they remain “augmented reality” and so, for the time being, a role remains for technology that offers real-time information. Intracardiac echocardiography (ICE) has more limited routine use in pediatric patients, though Headrick et al.^1^ found it to be safe with no related complications in pediatric and CHD procedures. Compared to controls, ICE use significantly reduced procedure duration, fluoroscopy exposure, and arrhythmia recurrence. While ICE improves outcomes, selective application is recommended to optimize its benefits in specific arrhythmia cases.

Coronary sinus (CS) accessory pathways (APs) remain challenging for even the most experienced operators as they may involve epicardial connections or be shielded by complex and atypical CS anatomy. To improve procedural outcomes, Drago et al.^2^ assessed the efficacy and safety of computed tomography (CT) scan-guided irrigated transcatheter ablation of APs in children, focusing on the CS region. Among 24 patients, the procedure achieved an 87.5% acute success rate with no complications, and no arrhythmia recurrences or adverse events occurred during a median follow-up of 15.1 months. The findings suggest that CT-guided EAM combined with irrigated radiofrequency ablation is a safe and effective approach for eliminating posterior septal and left posterior epicardial APs in children.

For left-sided radiofrequency catheter ablation (LCA) in pediatrics, significant practice variability exists regarding anticoagulation and discharge practices. Palmieri et al.^3^ evaluated the safety of same-day discharge and aspirin (ASA) use in pediatric patients after LCA. Among 376 patients, 89.9% were discharged the same day, and 20.2% were prescribed ASA, with a low rate of complications (2.7%)—primarily minor issues such as hematoma or headache. There was no correlation between complications and same-day discharge or ASA use, supporting the safety of same-day discharge without routine anticoagulation for pediatric LCA patients. Practice variation extends to routine intra- and post-procedural anticoagulation techniques as well, with Bhansali et al.^4^ conducting a survey of 73 pediatric and congenital electrophysiology studies (EPSs). This revealed that, pre-procedure, 25% discontinue ASA and 47% discontinue anticoagulants, while 32% administer heparin for all cases, with varied timing of the initial dose. Post-procedure, 58% do not use heparin infusions, and oral anticoagulants are typically started on the procedure day (34%) or the next day (53%), with low-dose ASA used by 74% for 4–6 weeks.

## Congenital heart disease: postoperative ablation, pulsed-field ablation, and tetralogy of Fallot non-invasive risk prediction

Kerr et al.^5^ presented a retrospective series of patients who underwent an EPS within 12 months of CHD surgery over a roughly two-decade period. Ablation was required in a very small subset of surgeries (0.1%) but showed an acute success rate of 82%, with manageable complications (8%) and a significant reduction in arrhythmia burden despite a 54% recurrence rate. The findings suggest that early postoperative ablation is a viable and effective option for managing arrhythmias in patients with complex surgical anatomy.

Pulsed-field ablation (PFA), a novel non-thermal energy source that has been received with great interest in adult electrophysiology, has not been extensively reported in pediatric or CHD patients. In a descriptive cohort review, Krause et al.^6^ evaluated its use for atrial fibrillation and atrial tachycardias in adult congenital heart disease (ACHD), demonstrating a 100% procedural success rate in isolating pulmonary veins and ablating arrhythmia substrates. Complications were minimal, with no irreversible events, but reversible coronary artery spasms were noted in one patient during cavotricuspid isthmus ablation. This spasm was resolved with intracoronary nitroglycerin after intravenous administration proved insufficient, highlighting the importance of preparing for coronary interventions during PFA near coronary structures. The findings suggest that PFA is a promising, safe, and effective alternative to thermal ablation, particularly for complex atrial substrates.

Kimura et al.^7^ described the use of three-dimensional late gadolinium enhancement cardiac magnetic resonance (3D-LGE-CMR) to non-invasively identify slow-conducting anatomical isthmuses (SCAIs) in patients with repaired tetralogy of Fallot (TOF), a key risk factor for ventricular tachycardia (VT). In two patient cohorts, 3D-LGE-CMR demonstrated high sensitivity (95%–100%) and specificity (90%–91%) in identifying SCAIs compared to invasive EAM, outperforming traditional risk scores. These findings suggest that 3D-LGE-CMR offers a highly accurate, non-invasive method for risk stratification and may optimize patient selection for preventive ablation. Similarly, Moore et al.^8^ investigated the role of multidetector computed tomography (MDCT) in evaluating anatomical isthmuses associated with sustained monomorphic VT in patients with repaired TOF. MDCT effectively identified relevant anatomical structures, including patch calcification and wall thickness, which correlated with isthmus conduction properties and ablation lesion distribution.

## Supraventricular tachycardia

Despite decades of clinical care, understanding adequate treatment for infants with supraventricular tachycardia (SVT) remains elusive. To examine the risk factors for readmission and outcomes in infants <6 months of age diagnosed with SVT, Vari et al.^9^ presented a cohort of 90 patients, in which 19 were readmitted within 31 days of discharge, with ventricular pre-excitation identified as a significant risk factor for early readmission. β-Blockers were the initial therapy in 66 and 28 patients who required a medication change. Readmitted infants required longer durations of anti-arrhythmic therapy and were more likely to undergo catheter ablation. The findings highlight the need for close monitoring of infants with ventricular pre-excitation to reduce readmission risk and improve outcomes.

## Innovative approaches: biological pacemakers and leadless pacing in small patients

Recent studies have examined biological methods for restoring cardiac conduction in animal models. Wolfson et al.^10^ developed a safer method to create biological pacemakers using synthetic TBX18 messenger RNA (mRNA), which reprograms heart cells to exhibit pacemaker activity while minimizing immune responses. Unlike adenovirus-based approaches, mRNA delivery localized expression to the heart, avoiding systemic spread and inflammation. In animal models, TBX18 mRNA provided adaptive cardiac pacing for up to 1 month, demonstrating its potential for safe and effective biological pacemaker development. Regarding current cardiac implantable electronic devices (CIEDs), the push has been for smaller leadless devices. The AVEIR™ (Abbott, Chicago, IL, USA) leadless pacemaker (LP) is now available in atrial and ventricular models. Rodriguez and Cortez^11^ reported on the placement of a 38-mm ventricular AVEIR™ LP in a 9-year-old, 23-kg patient with TOF, intermittent pacing needs, and a fractured ventricular pacing lead. This study demonstrates the implant feasibility of LPs in even the smallest of pediatric patients. From the same institution, English et al.^12^ described a case using an atrial AVEIR™ device for atrial pacing and a Micra™ device (Medtronic Inc., Minneapolis, MN, USA) for ventricular pacing in a 27-year-old ACHD patient with pacing dependence and ongoing concerns of infection. Long-term follow-up and extractability of these devices remain lacking, both in pediatric and adult patients. It is anticipated that 2025 will bring further studies regarding LPs, particularly as a dual-chamber pacing algorithm has been developed.

## Long-term outcomes and follow-up

Long-term follow-up data remain crucial to understanding the outcomes of CIEDs in pediatric patients with CHD. Hong et al.^13^ examined long-term lead survival in pediatric and CHD patients with pacemakers or defibrillators, finding survival rates of 87% at 10 years, 78% at 15 years, and 69% at 20 years. Greater somatic growth (≥5 cm/year) was the strongest predictor of lead failure, along with male sex, younger age, and epicardial lead implantation. CHD presence, lead insulation, and manufacturer did not affect lead longevity, emphasizing the need to account for growth-related factors in lead management. Clark et al.^14^ completed a Pediatric and Congenital Electrophysiology Society (PACES) study that assessed baseline compliance with CIED remote monitoring in pediatric and ACHD centers prior to a PACES-sponsored quality improvement initiative. While 77% of centers reported high remote monitoring enrollment, compliance with timely device transmissions and follow-ups varied by device type, with pacemakers and implantable cardiac monitors showing lower compliance rates. The PACES initiative aims to provide resources for improvement, with follow-up evaluations planned after quality improvement cycles.

## Cardiac resynchronization

Cardiac resynchronization therapy (CRT) in younger patients with or without CHD has struggled with the limitations of traditional non-invasive metrics like ejection fraction and QRS duration due to anatomical and surgical complexities. Menon et al.^15^ demonstrated that, after 1 year of CRT, patients showed clinical improvement and increased contractility, with significant enhancements in newer strain values such as global longitudinal strain and left atrial strain, despite minimal changes in traditional echocardiographic parameters. The findings suggest that strain-based measures provide a more accurate assessment of CRT response in this population than conventional metrics.
